# Improving Patient Engagement in Phase 2 Clinical Trials With a Trial-Specific Patient Decision Aid: Development and Usability Study

**DOI:** 10.2196/71817

**Published:** 2025-09-10

**Authors:** Iva Halilaj, Relinde Lieverse, Cary Oberije, Lizza Hendriks, Charlotte Billiet, Ines Joye, Brice Van Eeckhout, Anke Wind, Anshu Ankolekar, Philippe Lambin

**Affiliations:** 1 Department of Precision Medicine, Faculty of Health, Medicine and Life Sciences, Maastricht University, Universiteitssingel 40, Maastricht, 6229 ER, The Netherlands, 31 433883549; 2 Department of Internal Medicine, Catharina Ziekenhuis, Eindhoven, The Netherlands; 3 GROW - Research Institute for Oncology and Reproduction, Faculty of Health, Medicine and Life Sciences, Maastricht University, Maastricht, The Netherlands; 4 Department of Pulmonary Diseases, Maastricht University Medical Centre, Maastricht, The Netherlands; 5 Department of Radiation Oncology, University of Antwerp, Antwerp, Belgium; 6 Comunicare Solutions, Liege, Belgium

**Keywords:** patient decision aid, immunotherapy, L19-IL2, clinical trials, usability, informed decision-making, participative medicine

## Abstract

**Background:**

Making informed decisions about clinical trial participation can be overwhelming for patients due to the complexity of trial information, potential risks and benefits, and the emotional burden of a recent diagnosis. Patient decision aids (PDAs) simplify this process by providing clear information on treatment options, empowering patients to actively participate in shared decision-making with their doctors. While PDAs have shown promise in various health care contexts, their use in clinical trials, particularly in the form of trial-specific patient decision aids (tPDAs), remains underused.

**Objective:**

This study aims to address the challenge of patient comprehension of traditional clinical trial materials. We developed a freely accessible, user-friendly tPDA within the context of the ImmunoSABR phase 2 trial. The tPDA aimed to enhance informed decision-making regarding trial participation. The primary endpoint was usability, quantitatively measured by the System Usability Scale (SUS). Secondary endpoints included time spent on the tPDA, patient satisfaction ratings, and participants’ self-reported level of understanding of the trial.

**Methods:**

We developed the tPDA following the International Patient Decision Aid Standards and validated it through a structured, 3-phase iterative evaluation process. An initial evaluation was performed with 17 computer scientists who had expertise in biomedical applications, ensuring technical robustness. The content and usability were further refined through evaluations involving 10 clinicians and 8 medical students, focusing on clinical accuracy and user-friendliness. Finally, the tool was tested by 6 patients eligible for the ImmunoSABR trial to assess real-world applicability and patient-centered design.

**Results:**

Evaluations demonstrated the tPDA’s effectiveness in enhancing informed decision-making, directly addressing our primary end point of usability with an overall mean SUS score of 79.4 (SD 15.9), indicative of good usability. Addressing our secondary endpoints, patients completed the tPDA efficiently, with the majority (4/6) finishing in under 30 minutes, and all but 1 within 60 minutes. Qualitative feedback highlighted significant improvements in patients’ understanding of the trial details, reinforcing the tPDA’s role in facilitating better patient engagement and comprehension.

**Conclusions:**

Our study demonstrates the feasibility and potential of tPDAs to enhance patient comprehension and engagement in clinical trials. Integrating tPDAs offers a valuable addition to traditional paper-based and verbal communication methods, promoting informed decision-making and patient-centered care.

## Introduction

Cancer treatment has seen significant advancements in recent years, particularly in systemic therapy and radiation oncology [1]. These breakthroughs rely heavily on clinical trials to evaluate new therapies and improve patient outcomes, yet patient enrollment remains a persistent challenge [2]. The Institute of Medicine reports that 71% of phase 3 oncology trials fail to meet their enrollment goals [3,4]. This issue extends beyond academic trials, with around 80% of industry-sponsored trials also experiencing enrollment delays, potentially leading to substantial financial losses for drug developers [5]. In addition, the patient recruitment process is hindered by strict advertising regulations, the complexity of trial information, and external factors such as the COVID-19 pandemic [6]. Beyond financial and logistical concerns, low participation rates slow the translation of research findings into clinical practice, ultimately limiting patient access to novel treatments [7].

Several factors contribute to this underenrollment. Many patients misunderstand the purpose of clinical trials and overestimate the benefits of investigational treatments [8-10]. A meta-analysis of 103 studies showed that between 25% to 47% of clinical trial participants did not fully understand the implications of their participation, including its voluntary nature [11]. Only around half understood fundamental trial concepts, such as the role of placebos or the process of randomization. In addition, misconceptions persist among the general public, with some believing that trial participants may receive suboptimal care [12]. Even when eligible patients are identified, they often struggle to navigate the lengthy, complex, and technical language commonly found in patient information forms and trial documentation [13]. These findings indicate that comprehension is a considerable barrier to enrollment, and there is a critical need for tools that support patients’ understanding and informed consent in clinical trials.

Patient decision aids (PDAs) have been widely studied as tools that support shared decision-making by providing unbiased information about treatment options, helping patients clarify their values, and supporting communication between patients and clinicians [14-17]. These tools are becoming increasingly relevant in oncology as the complexity of treatment options increases and decisions often involve significant trade-offs between treatment efficacy and quality of life [18]. While typically used for clinical treatment decisions, their application in the context of clinical trial enrollment remains underexplored. Unlike standard PDAs, trial-specific PDAs (tPDAs) address the unique complexities of trial participation, yet little research has examined their effectiveness in this setting. A 2015 Cochrane review found limited evidence on tPDA efficacy, citing a scarcity of relevant studies and inconclusive results regarding knowledge, decisional conflict, and trial participation. Although some evidence suggested that tPDAs might reduce decisional regret, key factors such as risk perception and value-based decision-making remained unexplored.

Despite the growing body of research on tPDAs since the 2015 review, knowledge gaps persist regarding their optimal design and implementation for complex clinical trials, particularly those investigating novel therapies. Given the high cognitive burden associated with trial decision-making, tPDAs could play a critical role in improving patient comprehension and supporting informed consent. Our study addresses this need by developing and evaluating an interactive tPDA for the ImmunoSABR trial, a phase 2 trial investigating a novel combination therapy (NCT03705403) [19]. This multicenter, randomized, phase 2 trial across 15 centers in 6 European countries investigated the efficacy of combining stereotactic radiotherapy and immunotherapy in prolonging progression-free survival while minimizing toxicity and preserving quality of life for patients with non–small cell lung cancer (NSCLC). However, recruitment was hindered by the complexity of trial-related information, with patients struggling to interpret the detailed eligibility criteria, treatment sequences, and potential risks and benefits. Standard patient information documents, even when supplemented with visual aids, often proved inadequate in conveying the complexities of the ImmunoSABR trial, including its novel immunotherapy-radiotherapy combination and its implications. Recognizing this, we developed the tPDA as a supplementary tool to enhance patient comprehension and facilitate informed decision-making regarding trial participation [20]. This paper describes our rigorous, multistakeholder evaluation process, providing evidence for the tPDA’s potential to enhance patient comprehension, facilitate informed decision-making, and foster active engagement in the clinical trial process.

## Methods

### Overview

The tPDA’s development and validation followed a structured, multiphase approach, incorporating feedback from diverse stakeholders: computer scientists with expertise in medical applications, clinicians and medical students from hospitals in the Netherlands and Belgium, and Dutch patients with stage 4 NSCLC eligible for the ImmunoSABR trial. Each evaluation phase engaged these groups in distinct ways to ensure a comprehensive assessment of the tPDA. The tPDA was developed and tested locally with a subset of eligible patients, rather than being deployed across all ImmunoSABR trial sites, as a broader rollout would have required additional ethical approvals, validated translations and back translations in various languages, and logistical coordination beyond the scope of this study.

### Recruitment

We used a purposive sampling approach to select participants based on their relevance to the tPDA evaluation. The groups and their roles were as follows:

1. Computer scientists (n=18): individuals from Maastricht University with expertise in medical applications were selected to assess the tPDA’s technical aspects, user interface, and information visualization.
2. Clinicians and medical students (n=18): 10 clinicians and 8 medical students, recruited from hospitals in the Netherlands and Belgium, evaluated the tPDA’s content accuracy, usability, and comprehensibility from a health care professional’s perspective.
3. Potential trial participants (n=6): Dutch patients with stage 4 NSCLC, eligible for the ImmunoSABR trial, participated in the final evaluation phase to assess the tPDA’s usability and comprehensibility from a patient’s perspective. Patients eligible for usability testing were required to meet the same inclusion and exclusion criteria as participants in the ImmunoSABR phase 2 trial (Clinicaltrials.gov: NCT03705403). These criteria were stringent, including limitations on metastatic burden, specific organ function thresholds, and performance status requirements.

### Development of the tPDA

**Figure 1.**
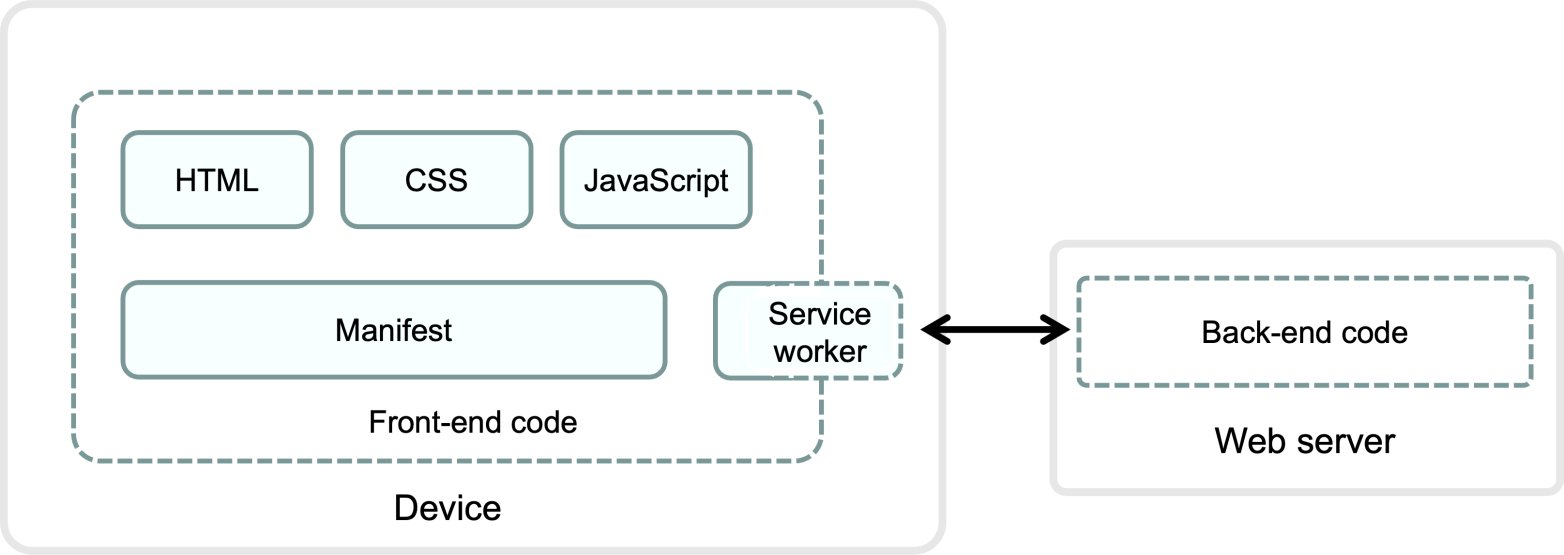
High-level architecture of the Progressive Web App used for the trial-specific patient decision aid.

The initial tPDA prototype was developed adhering to the International Patient Decision Aid Standards, complemented by clinician consultations and a comprehensive literature review. To ensure optimal user-friendliness and accessibility, we designed the tPDA as a Progressive Web App (PWA) using Firebase [21]. This approach allows the tPDA to function seamlessly across various platforms, including desktop and mobile devices, using any standards-compliant browser. The front-end development leveraged JavaScript, HTML5, CSS3, and a JSON manifest. The PWA’s high-level architecture, illustrated in Figure 1, involves a web server hosting the back-end code (written in Node.js) and the user’s device running the front-end code, including the service worker, manifest, HTML, CSS, and JavaScript components. The tPDA’s development then proceeded through a phased approach, incorporating multiple rounds of evaluation and refinement based on feedback from the diverse stakeholder groups.

### Evaluation Instruments

To gather feedback from participants in each round of evaluation, a voluntary questionnaire was introduced. This questionnaire consisted of 17 items, including 10 questions adapted from the validated System Usability Scale (SUS) [22,23]. These questions, translated into Dutch, aimed to evaluate the tPDA’s performance, potential value, and user satisfaction, considering factors such as comprehensibility, usability, and the perceived value of the information. The tPDA’s effectiveness and efficiency in supporting decision-making were also assessed. Responses to the SUS questions were recorded on a 5-point Likert scale. The remaining 7 questions were open-ended and designed to gather qualitative feedback on aspects such as the user’s experience with the app, the time taken to complete it, and suggestions for improvement.

### Prototype Evaluation

#### Round 1: Computer Scientist Review

The initial tPDA prototype was evaluated by 17 computer scientists, focusing on technical functionality, information presentation, and visual design. Their feedback informed the development of the second prototype, which featured improved logical sequencing and enhanced visual representations.

#### Round 2: Clinicians and Medical Students Review

The second tPDA prototype was evaluated by 10 clinicians and 8 medical students. Their assessment focused on the accuracy of the information presented, overall usability, and comprehensibility. Feedback from this round was incorporated into the development of the third prototype.

#### Round 3: Final Review and Potential Trial Participants’ Feedback

**Figure 2.**
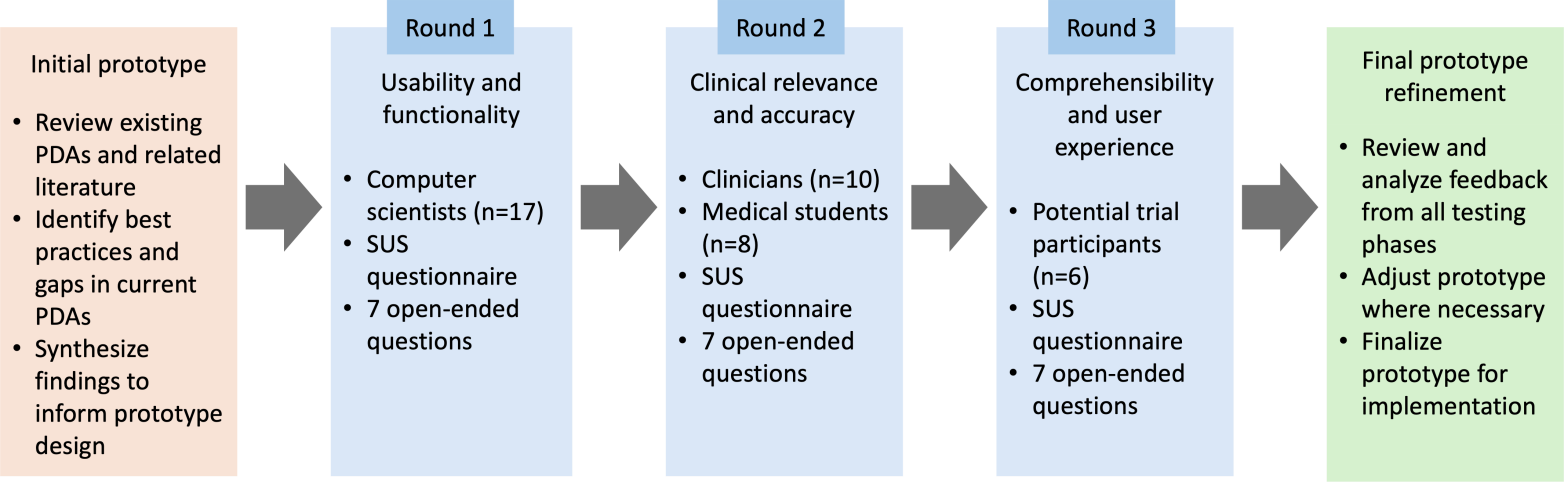
The iterative development and evaluation process of the trial-specific patient decision aid, incorporating feedback from computer scientists, clinicians, and potential trial participants. PDA: patient decision aid; SUS: System Usability Scale.

The final tPDA version underwent evaluation by potential ImmunoSABR trial participants. Their feedback focused on the app’s comprehensibility, usability, and the perceived value of the information presented. This crucial input provided insights into the tPDA’s real-world impact on the decision-making process for potential trial participants. Figure 2 summarizes the full development and evaluation process. The full set of evaluation materials, including the SUS questionnaire, open-ended patient responses, descriptive statistics, and prototype examples, is available in Multimedia Appendix 1.

### Data Analysis

Adhering to standardized SUS methodology, raw scores (0‐40) were converted to a 0‐100 scale and ranked in percentiles for easier interpretation [22]. Given the tPDA’s specific purpose for one-time trial information rather than regular use, we omitted the SUS item “I will use this app frequently.” A recent study indicates that as long as the multiplier is adjusted appropriately (from 2.5 to 2.7), this will not significantly affect the final scores [24]. Data analysis was performed using IBM SPSS 29.0.1. Qualitative feedback collected through open-ended survey responses was used to complement usability metrics. Given the brevity of responses, no formal thematic analysis was conducted, as we did not use structured qualitative data collection methods, such as interviews or focus groups. Instead, responses were reviewed descriptively to identify usability concerns and guide iterative improvements.

### Ethical Considerations

The study was conducted in compliance with the ICH Harmonized Tripartite Guideline for Good Clinical Practice and received ethical approval from the medical ethics committee of Maastricht University Medical Centrum on September 19, 2021 (approval number: NL67629.068.18). All patients participating in the ImmunoSABR trial provided oral and written informed consent after receiving comprehensive information about the study. While informed consent was mandatory for participation in the ImmunoSABR trial, it was not required for using the tPDA web app, which was tested as part of the trial’s broader evaluative processes. Participant data were anonymized and stored on a secure server within the lab of the coordinating researchers. Participants were not compensated for taking part in the study.

## Results

### Overview

A total of 41 individuals participated in the evaluation of the tPDA across 3 rounds. This included 17 computer scientists, 10 clinicians, 8 medical students, and 6 potential trial participants. The majority of the clinicians were female (14/18, 77.8%) and aged less than 30 years (10/18, 55.6%). Among the clinicians, radiation oncologists constituted the largest group (5/18, 27.8%), followed by medical oncologists (3/18, 16.7%). The medical students had less than 10 years of experience and clinicians between 10 and 20 years of experience. Full demographic and clinical characteristics of the clinicians and medical students are presented in Table 1.

**Table 1.** Demographic characteristics of clinician participants (n=18).

Characteristics	Participants
Age (years), n (%)	
<30	10 (55.6)
30‐49	6 (33.3)
50‐65	2 (11.1)
Gender, n (%)	
Male	4 (22.2)
Female	14 (77.8)
Specialization, n (%)	
Pulmonologist	1 (5.6)
Radiation oncologist	5 (27.8)
Medical oncologist	3 (16.7)
General practitioner	1 (5.6)
Medical student	8 (44.4)
Experience (years), n (%)	
<10	10 (55.6)
10‐19	6 (33.3)
20‐29	2 (11.1)

### Overall Usability Assessment and Prototype Evolution

**Figure 3.**
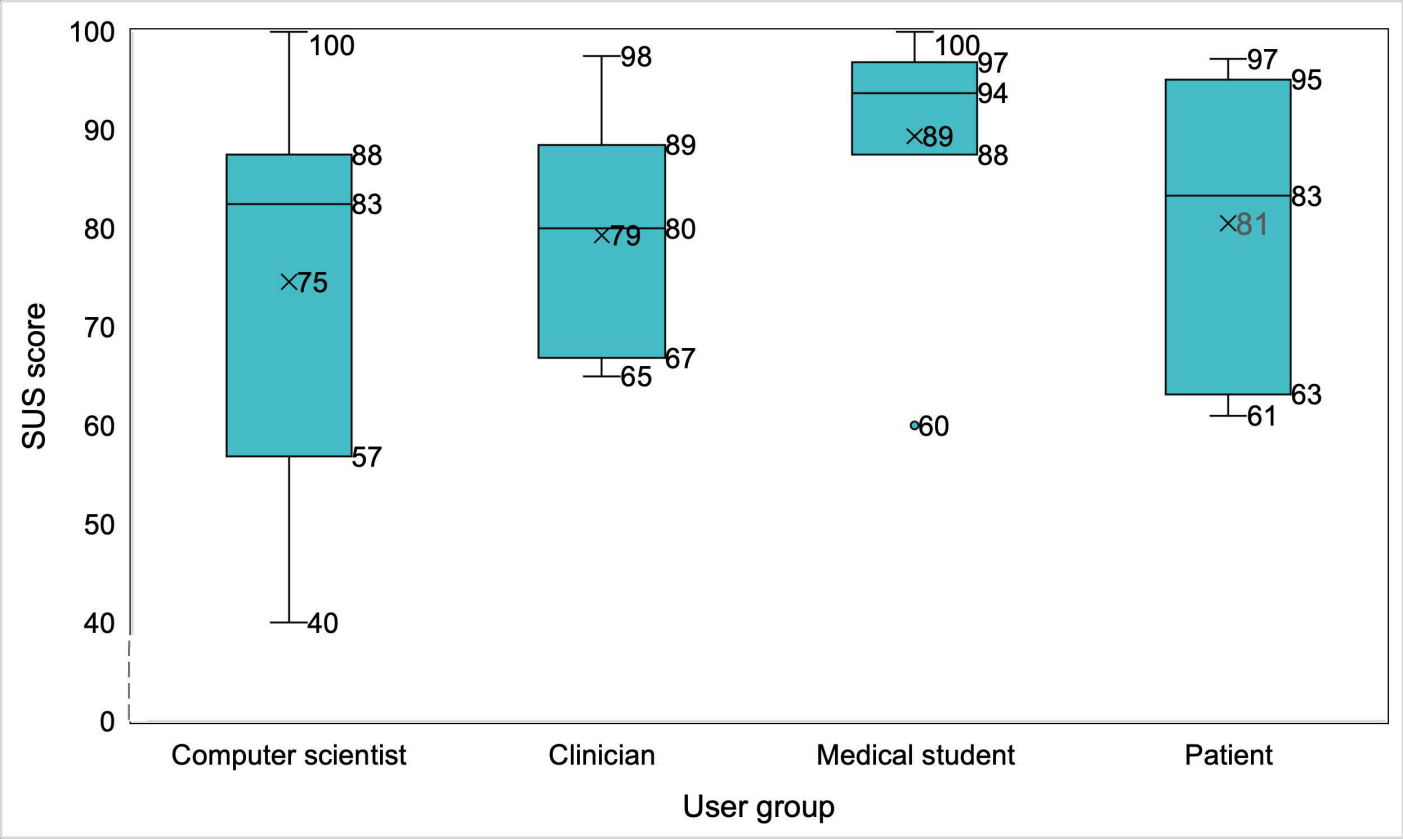
The IQR, median, and mean (denoted by “x”) of the SUS scores from different participant groups across successive iterations of the tPDA. Scores reflect independent usability assessments rather than a longitudinal comparison. tPDA: trial-specific patient decision aid; SUS: System Usability Scale.

Across all evaluation rounds and participant groups, the tPDA demonstrated consistently favorable usability, as evidenced by the SUS scores (Figure 3). It is important to note that SUS scores were collected during iterative testing phases, with different stakeholder groups evaluating successive versions of the tPDA. As different groups participated at each stage, these scores reflect independent assessments rather than direct comparisons of usability improvements over time. The final prototype garnered a commendable overall SUS score of 79.4 (SD 15.9), placing it within the 85th-89th percentile range. Notably, patients, the intended end users, exhibited particularly high satisfaction, with a median SUS score falling in the “excellent” range. Medical students also expressed strong endorsement, with an average SUS score of 89.4 (SD 12.7). Clinicians (n=10) generally provided a favorable assessment, with an average SUS score of 79.3 (SD 11, 85th-89th percentile). This positive reception was particularly pronounced among radiation oncologists (n=5), who expressed high satisfaction with an SUS score of 80.5 (SD 11.6). Medical oncologists (n=3) and the general practitioner (n=1) also provided positive evaluations, with SUS scores of 80.8 (SD 13.8) and 80, respectively. In contrast, the pulmonologist (n=1) gave a lower score of 68.

**Figure 4.**
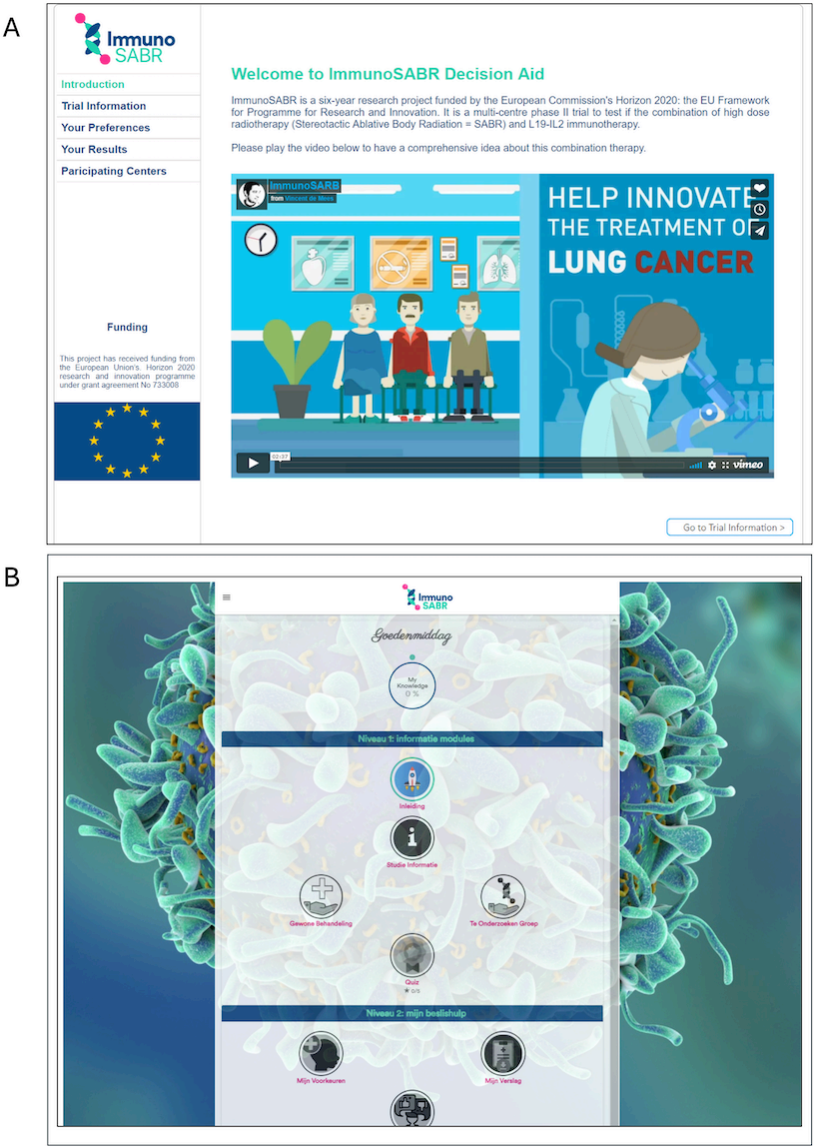
Evolution of the trial-specific patient decision aid interface: (**A**) initial prototype and (**B**) final prototype after incorporating feedback from multiple evaluation rounds.

The iterative refinement process led to significant improvements in the tPDA’s interface and user experience. Figure 4 showcases both the initial prototype and the final version, highlighting the visual and functional enhancements achieved through incorporating feedback from various stakeholders.

### Iterative Refinement and Feedback

#### Round 1

The initial prototype was first evaluated by 17 computer scientists. Beyond the positive SUS scores, computer scientists provided constructive feedback, highlighting areas for improvement. These included the app’s limited one-time use, cross-device interface inconsistencies, typos and unclear or overlapping text, and the lack of hover features and clear visual cues for clickable elements. These insights guided the development of the second prototype, with a focus on enhancing logical sequencing and visual representations.

#### Round 2

Incorporating feedback from the first round, the second prototype underwent evaluation by 10 clinicians and 8 medical students. While the core interface remained largely unchanged, refinements were made to enhance usability and address technical issues. Clinicians and medical students provided feedback on several key themes. They advocated for broader language accessibility, suggesting the translation of videos and potentially other content into multiple languages. In addition, they recommended refining textual content for enhanced clarity and providing further elaboration on specific topics to improve patient understanding. Ensuring accuracy and clarity in animation subtitles was also emphasized. Furthermore, they recommended enhancing the system’s ability to save user input, preventing data loss upon refresh. Specific suggestions, such as incorporating a flowchart illustrating the experimental therapy timeline, aimed to reduce reliance on dense text and improve patient comprehension of complex information. This feedback informed the development of the third prototype, with a focus on addressing these areas and further refining the tPDA’s content and user experience. Following these improvements, the final tPDA prototype was evaluated by potential trial participants.

#### Round 3

The final tPDA prototype was evaluated by 6 potential ImmunoSABR trial participants. Their feedback was overwhelmingly positive, with an average SUS score of 81, placing it in the 90th-95th percentile range. Qualitative feedback revealed that 5 out of 6 patients completed the tPDA within 60 minutes, with 4 finishing in under 30 minutes. The tPDA received high ratings, with 4 patients giving it a 9 out of 10 and 1 awarding a perfect 10. Notably, no patients identified any missing topics, indicating that the tPDA adequately covered the necessary information. Patients specifically praised the tPDA’s logical structure, clarity, comprehensibility, and the inclusion of an informative video. Despite the positive feedback, some areas for improvement were also identified. One patient expressed that their interest in trying a new medication was misinterpreted as a potential reason for not participating in the trial, suggesting a need for clearer phrasing in certain questions. Another patient suggested refining 2 questions to offer more nuanced response options.

## Discussion

### Overview and Principal Findings

While clinical trials are crucial for driving progress in cancer treatment, the process of informing and recruiting eligible patients presents persistent challenges [25,26]. Traditional patient information forms, often dense and filled with technical jargon, can impede comprehension and hinder informed decision-making [27]. This gap in effective communication not only jeopardizes patient safety but also raises ethical concerns regarding the validity of informed consent. Recognizing the need for innovative solutions, our study focused on developing and evaluating a digital tool designed to enhance patients’ understanding of the ImmunoSABR trial and help them make an informed choice on whether to participate.

Our study provides promising evidence that the ImmunoSABR tPDA enhances patient comprehension and engagement in the clinical trial decision-making process. The usability evaluation results suggest that the tPDA effectively enhances patient comprehension, with positive feedback highlighting its structured format, visual aids, and interactivity as key strengths. The variability in time required to complete the tPDA (with 2 participants requiring up to 60 min) suggests that the tPDA’s length may be a limiting factor in certain clinical settings where patients have restricted availability. Future iterations may explore options to streamline content delivery while preserving key decision-making information. Presenting information in an interactive and visually appealing format could empower patients to take ownership of their health care choices, aligning with the broader trend toward patient empowerment and shared decision-making in health care [28]. The combination of visual aids and interactive questions to check comprehension within the tPDA actively supports this patient-centered approach to learning and decision-making [29]. These findings align with broader efforts to enhance patient comprehension in clinical trials. Prior studies that explored the impact of tPDAs across different trial settings offer valuable context for interpreting our results.

### Context Within Prior Research

#### tPDAs in Decision-Making

Previous research has explored the use of tPDAs in various clinical contexts, highlighting improvements in patient knowledge, reduced decisional regret, and increased willingness to participate in trials [30-39]. These studies, ranging from breast cancer prevention to pediatric oncology, have shown that tPDAs can significantly enhance patient comprehension and facilitate informed decision-making by presenting complex trial information in a clear, concise, and accessible manner. However, most prior studies have primarily focused on measuring knowledge retention and decisional outcomes, rather than assessing the usability of these tools across different stakeholder groups.

Our findings build on this body of evidence by shifting the focus toward usability and patient experience in the context of a phase 2 trial investigating a novel combination therapy. Unlike previous work, which primarily measured knowledge retention or decisional regret, our study systematically evaluated the feasibility of implementing a tPDA through multistakeholder usability testing. We incorporated feedback from patients, clinicians, and computer scientists in a user-centered design process, thereby aligning with best practices in digital health intervention development, emphasizing the importance of collaborative and iterative design [36]. This approach ensures that tPDAs are not only informative but also accessible, engaging, and well integrated into clinical workflows.

#### Uncertainty of tPDA Impact on Trial Enrollment

While our study indicates that participants perceived the tPDA as helpful in structuring trial-related information in a clear and accessible manner, we did not directly measure its effect on patient decision-making or enrollment rates. The influence of tPDAs on trial participation remains unclear, as previous studies have reported mixed findings. Although some evidence suggests that tPDAs support better decisional alignment with patient values, they do not necessarily lead to higher enrollment rates [32,37,38]. In fact, the only study that measured an increase in trial participation (20.6% vs 9%) did not find statistical significance [39]. This suggests that while tPDAs improve comprehension and decisional confidence, they may not overcome other barriers to enrollment, such as concerns about treatment burden, logistical constraints, or mistrust in research. Future studies should incorporate qualitative methods, such as semistructured interviews or focus groups, to explore how tPDAs influence patient decision-making beyond information delivery. This would provide deeper insights into patient motivations and help refine these tools for greater impact.

#### Balancing Standardization and Adaptability

A key advantage of tPDAs, highlighted both in our findings and in prior work, is their potential for standardizing information delivery across different trials and health care settings. However, achieving true standardization presents challenges, as clinical trials vary widely in treatment complexity, eligibility criteria, and patient demographics [40,41]. Ensuring that tPDAs remain customizable while maintaining core principles of informed decision-making is essential. Future research should explore adaptable tPDA frameworks, allowing for modular content that can be tailored to specific trial protocols while preserving a consistent approach to patient education. This would enable widespread adoption while ensuring that patient needs remain at the center of tPDA design.

#### Scalability and Cost

The scalability of tPDAs remains a pressing issue, particularly in resource-limited settings where patients may face digital literacy barriers, socioeconomic disadvantages, or language constraints. While web-based tPDAs offer broad accessibility, their effectiveness depends on ensuring equitable access across diverse patient populations. This includes translating tPDAs into different languages in international trials. Future research should examine the feasibility of alternative formats, such as printed materials, audio-visual presentations, or interactive decision aids facilitated by health care providers. Evaluating the comparative effectiveness of digital versus nondigital tPDAs in larger, more diverse cohorts, including older patients and those from underrepresented backgrounds, would provide critical insights into how best to implement these tools across different health care environments [42,43]. A randomized study or A/B testing approach could provide valuable insights into whether tPDAs lead to measurable improvements in patient comprehension, decision-making confidence, and trial enrollment rates compared to conventional materials.

Ensuring equitable access across diverse patient populations is crucial for the successful adoption of tPDAs at scale. This could involve exploring alternative formats, such as printed materials, video-based decision aids, or clinician-facilitated interactive tools. In addition, modifications to digital tPDAs, such as simplified navigation, larger fonts, audio narration, and adaptive content tailored to users’ digital proficiency, could enhance usability and inclusion. Guided sessions, where health care professionals, such as clinicians or nurses, assist patients in using digital decision aids, may further support patients with limited health literacy or digital experience [44,45]. Addressing these factors is essential to maximizing the impact of tPDAs and ensuring that they remain accessible, effective, and patient-centered as adoption increases.

Finally, the cost-effectiveness of tPDAs remains an area of ongoing investigation. While some studies suggest that these tools may lead to long-term cost savings by improving treatment adherence and reducing decisional regret, the economic impact of tPDAs has not been widely assessed [39,46,47]. Developing and implementing these tools requires investment in software development, clinical integration, and continuous evaluation. Standardized methodologies for assessing cost-effectiveness should be prioritized in future research, along with innovative funding models, such as publicly accessible platforms or partnerships between research institutions and industry sponsors, to address financial barriers to widespread implementation.

### Limitations

#### Timing and Recruitment Constraints

While our study offers valuable insights into the potential of tPDAs in clinical trials, it is essential to acknowledge its limitations. One major constraint was the timing of the tPDA’s development, which coincided with the active patient recruitment phase of the ImmunoSABR trial. By the time the tPDA received ethical approval and was introduced to Dutch trial sites, a significant number of participants had already been enrolled, limiting its immediate impact. This highlights a missed opportunity for earlier patient engagement. However, ensuring that the tPDA was functionally and medically sound before exposing vulnerable patients to its content was a necessary ethical consideration. Digital health interventions designed for clinical decision-making require rigorous validation to prevent misinformation or undue patient burden. We addressed this by following an iterative usability process, prioritizing early validation with clinicians and computer scientists before introducing the tPDA to patients. Future studies should prioritize parallel development of tPDAs alongside clinical trial protocols, allowing for timely implementation at the start of patient recruitment.

Another limitation was the modest response rate among trial participants, despite proactive engagement efforts through trial nurses. Several factors likely contributed to this, including the strict eligibility criteria of the ImmunoSABR trial, which restricted usability testing to a narrow patient population. In addition, the cognitive and logistical demands of trial participation may have affected patients’ willingness to engage with an additional decision aid. Given these constraints, our study emphasized usability testing with clinicians and computer scientists in the earlier development phases to refine the tPDA before patient testing. Future studies could enhance the generalizability of findings by recruiting a broader sample of patients with NSCLC, including those not actively considering trial participation, to assess tPDA usability in a wider clinical context.

#### Data Tracking Limitations

The lack of systematic tracking of patient engagement presents another limitation. The exact number of patients approached remains undetermined, preventing a precise calculation of engagement rates and a comprehensive analysis of participation barriers. While we speculate that some patients may have accessed the tPDA without completing the embedded evaluation questionnaire, the tPDA’s privacy-centric design, which avoids storing patient data beyond submitted responses, limits our ability to verify this assumption. In addition, the absence of a separate informed consent process for tPDA usage may have affected how patients perceived its role in their decision-making. Clinic understaffing may have further hindered tPDA promotion and patient support. To address these issues, future studies should implement structured tracking methods to capture patient engagement metrics, including the number of patients informed about the tPDA, actual usage statistics, and completion rates. Such data would provide a more nuanced understanding of patient engagement and identify areas for improving tPDA implementation and support.

#### Generalizability and Accessibility Challenges

Finally, the generalizability of our findings requires further consideration. The SUS scores reflect perspectives from diverse user groups, including medical students, clinicians, and patients. While clinicians and medical students may have evaluated usability through a clinical and interface design lens, patients provided direct end-user feedback, potentially leading to variation in usability perceptions based on professional experience and digital literacy. Future research should further examine these differences, ensuring that usability assessments prioritize patient-centered evaluations. In addition, our study focused on a digital tPDA, which may overlook accessibility challenges faced by patients with limited technological proficiency. Older patients, who represent a significant proportion of the NSCLC patient population, may encounter difficulties navigating web-based tools [48]. Socioeconomic disparities also present barriers to digital tool adoption, as some patients may lack access to reliable internet or digital devices [49].

Despite these limitations, our findings provide valuable insights into the usability of tPDAs in clinical trial settings. Addressing these challenges, along with the broader research directions outlined in this discussion, will be critical to ensuring that digital decision aids remain accessible, effective, and widely applicable.

### Conclusions

Our study highlights the feasibility of implementing the ImmunoSABR tPDA to support patient comprehension and informed decision-making in clinical trials. The tPDA was well-received, with usability assessments reflecting a strong user experience (SUS score of 79.4), aligning with established thresholds for high usability. Moreover, the majority of participants were able to complete the tPDA in under 30 minutes, showcasing its efficiency in delivering essential trial information swiftly.

The tPDA offers a patient-centered approach to clinical trial education by addressing the limitations of traditional information provision methods and using interactive visual elements. While it has shown promising results in enhancing immediate patient engagement, further research is needed to fully explore its impact on trial enrollment and long-term patient outcomes. The initial positive evaluation results and alignment with existing literature suggest that tPDAs represent a valuable tool for empowering patients and promoting ethical clinical research practices.

Future efforts should focus on addressing implementation challenges, ensuring accessibility for diverse patient populations, and developing standardized yet adaptable tPDA frameworks to facilitate their wider adoption. This will maximize their benefits for both patients and clinical research, potentially leading to improved trial participation rates and more informed patient consent.

## Supplementary material

10.2196/71817 Multimedia Appendix 1 Full set of evaluation materials, including the System Usability Scale questionnaire (English and Dutch), trial participants’ open-ended responses, descriptive statistics of System Usability Scale scores, an example of a values clarification question, and a sample patient PDF report generated by the trial-specific patient decision aid.

## Abbreviations

NSCLC: non–small cell lung cancer
PDA: patient decision aid
PWA: Progressive Web App
SUS: System Usability Scale
tPDA: trial-specific patient decision aid
