# Supplementary material for: Improving Patient Engagement in Phase 2 Clinical Trials With a Trial-Specific Patient Decision Aid: Development and Usability Study
Source: J Med Internet Res. 2025 Sep 10;27:e71817. doi: 10.2196/71817 (PMC12422530; doi:10.2196/71817)
Supplement: Multimedia Appendix 1 [file jmir-v27-e71817-s001.doc]

**Table S1.** Overview of questions in the SUS questionnaire in English and Dutch.

| **English SUS question** | **Dutch (translated) SUS question** |
| --- | --- |
| 1. I have already received a substantial amount of information regarding the study from both the nursing staff and written materials. This application has assisted me in making a decision regarding my participation in the research. 2. I found the system unnecessarily complex. 3. I thought the system was easy to use. 4. I think that I would need the support of a technical person to be able to use this system. 5. I found the various functions in this system were well integrated. 6. I thought there was too much inconsistency in this system. 7. I would imagine that most people would learn to use this system very quickly. 8. I found the system very cumbersome to use. 9. I felt very confident using the system. 10. I needed to learn a lot of things before I could get going with this system. | 1. Ik heb al veel informatie over de studie gekregen van de verpleegkundigen en op papier. Deze app heeft mij geholpen om een beslissing te maken of ik mee wil doen aan het onderzoek. 2. Ik vond de app ingewikkeld. 3. Ik vond de app makkelijk te gebruiken. 4. Ik had hulp nodig van een technisch persoon om deze app te kunnen gebruiken. 5. Ik vond de app in zijn geheel goed werken. 6. Ik vond dat er te veel tegenstrijdigheden in de app zaten. 7. Ik denk dat de meeste mensen snel kunnen omgaan met deze app. 8. Ik vond de app omslachtig in gebruik. 9. De app voelde tijdens gebruik vertrouwd aan. 10. Ik gebruik zelden apps, daarom heb ik moeten leren hoe ik deze app moest gebruiken. |
| **Open questions** | |
| 1. Which device did you utilize for the application? 2. How much time did you require to navigate through the application? (<30min; 30-60 min; >60min) 3. On a scale from 1 (poor) to 10 (excellent), how would you rate the application? 4. Is there a subject that you found lacking within the application? 5. Which aspects of the application did you find pleasing? 6. Would you suggest any alterations to the application; if so, what changes would you propose? 7. Do you have any additional comments? | 1. Welk apparaat heb je gebruikt? 2. Hoeveel tijd had u nodig om door de app te gaan? (<30min; 30-60 min; >60min) 3. Op een schaal van 1 (slecht) tot 10 (uitstekend), hoe zou u de app beoordelen? 4. Is er een onderwerp dat u hebt gemist in de app? 5. Welke aspecten van de app beviel u? 6. Zou u iets in de app willen veranderen en zo ja, wat? 7. Heeft u nog aanvullende opmerkingen? |

Table S2: Answers to open questions from trial participants

| **Open question** | **Answer** |
| --- | --- |
| Which device did you utilize for the application? | “I had to search for where I could click, as there was no hand icon on the cursor. However, after the first item, that was fine, of course.” |
| How much time did you require to navigate through the application? (<30min; 30-60 min; >60min) | 4x “<30 min”; 1x “30-60 min” |
| On a scale from 1 (poor) to 10 (excellent), how would you rate the application? | 4x “9”; 1x “10” |
| Is there a subject that you found lacking within the application? | 5x “No” |
| Which aspects of the application did you find pleasing? | “The logical sequence of all components.”  “Everything presented clearly in a row.”  “Clear and comprehensible information.”  “It is logically structured.”  “The video.” |
| Would you suggest any alterations to the application; if so, what changes would you propose? | “I responded that I would find testing a new medication exciting, and this emerged as a counterargument in the 'outcome'. However, I disagree with this; while I do find it exciting, it would not be a reason for me not to participate.”  “For Questions 4 and 5, I could only provide an answer with which I was not entirely in agreement. While I am willing to participate in a study involving a new medication, I naturally expect that there is a significant likelihood of benefiting from it. In Question 7, the sentence is incorrect, as a word is missing” |
| Do you have any additional comments? | 5x “No” |

*Table S3: Descriptive statistics of the SUS scores for each participant group.*

| **Participant group** | **Mean** | **Max** | **Min** | **Std dev** |
| --- | --- | --- | --- | --- |
| Computer Scientist | 83 | 100 | 57 | 18.4 |
| Physicians | 79 | 99 | 65 | 11.8 |
| Medical Students | 94 | 100 | 88 | 12.7 |
| Patients | 81 | 97 | 61 | 13.1 |


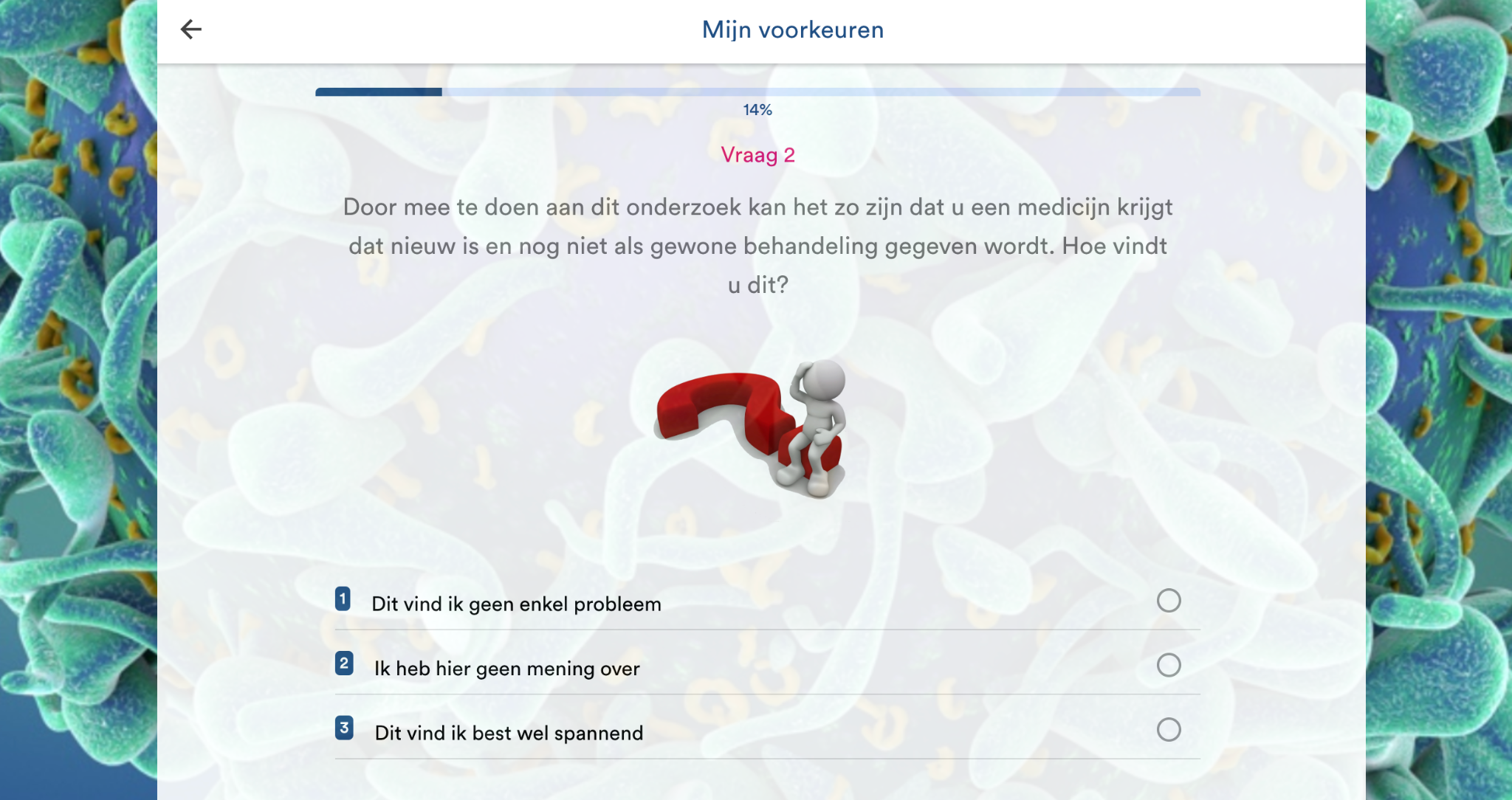


Figure S4: An example of a values clarification question that helps the patient understand their personal preferences in the context of the study. In this case, the patient is being informed that by participating in this study, there is a possibility that they will receive a new medicine that is currently not offered as a standard treatment. The patient is then asked to evaluate how they feel about this information, to which they can answer “I don’t find that a problem”, “I do not have an opinion about this”, or “I find that rather stressful”.

| 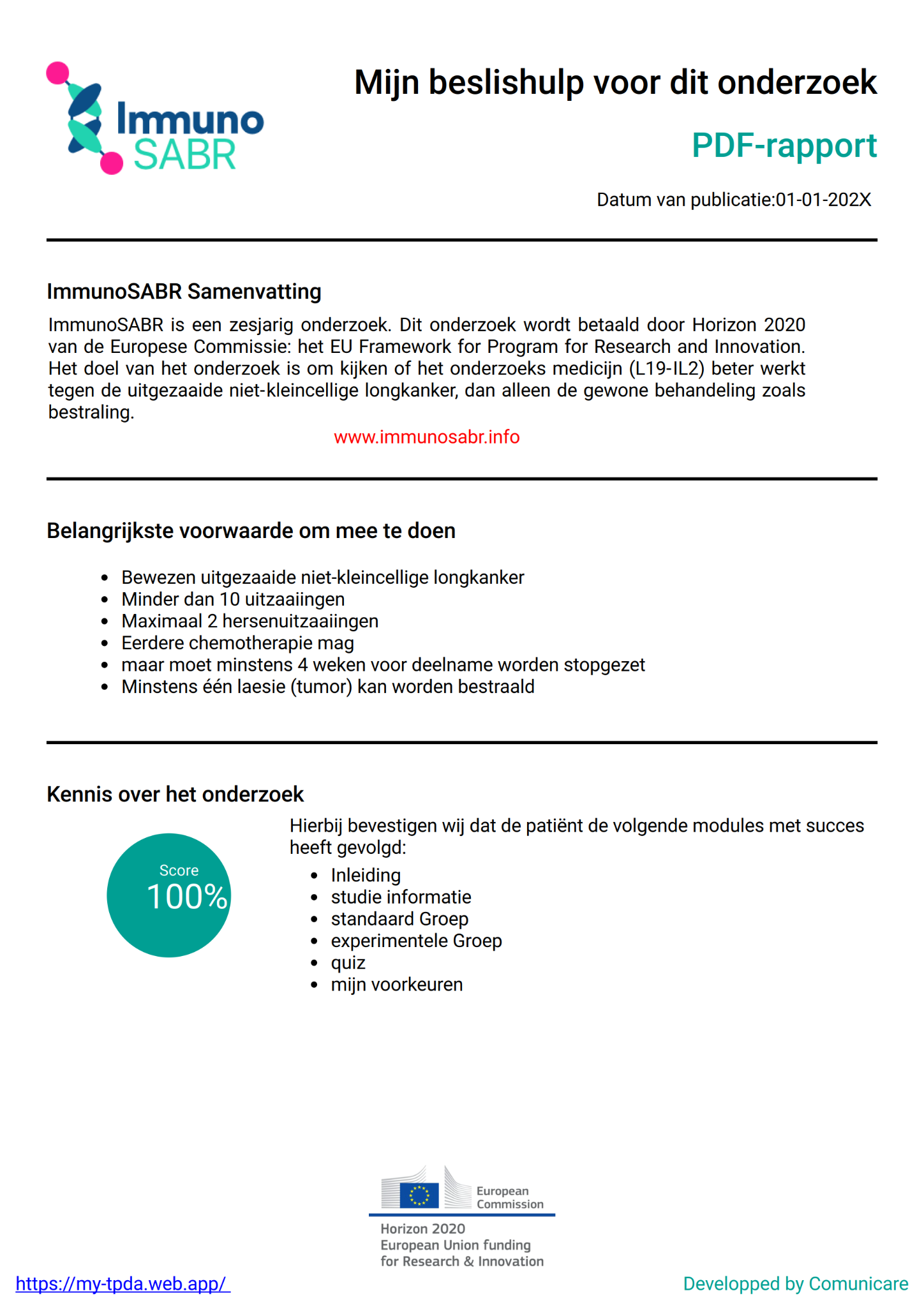 | 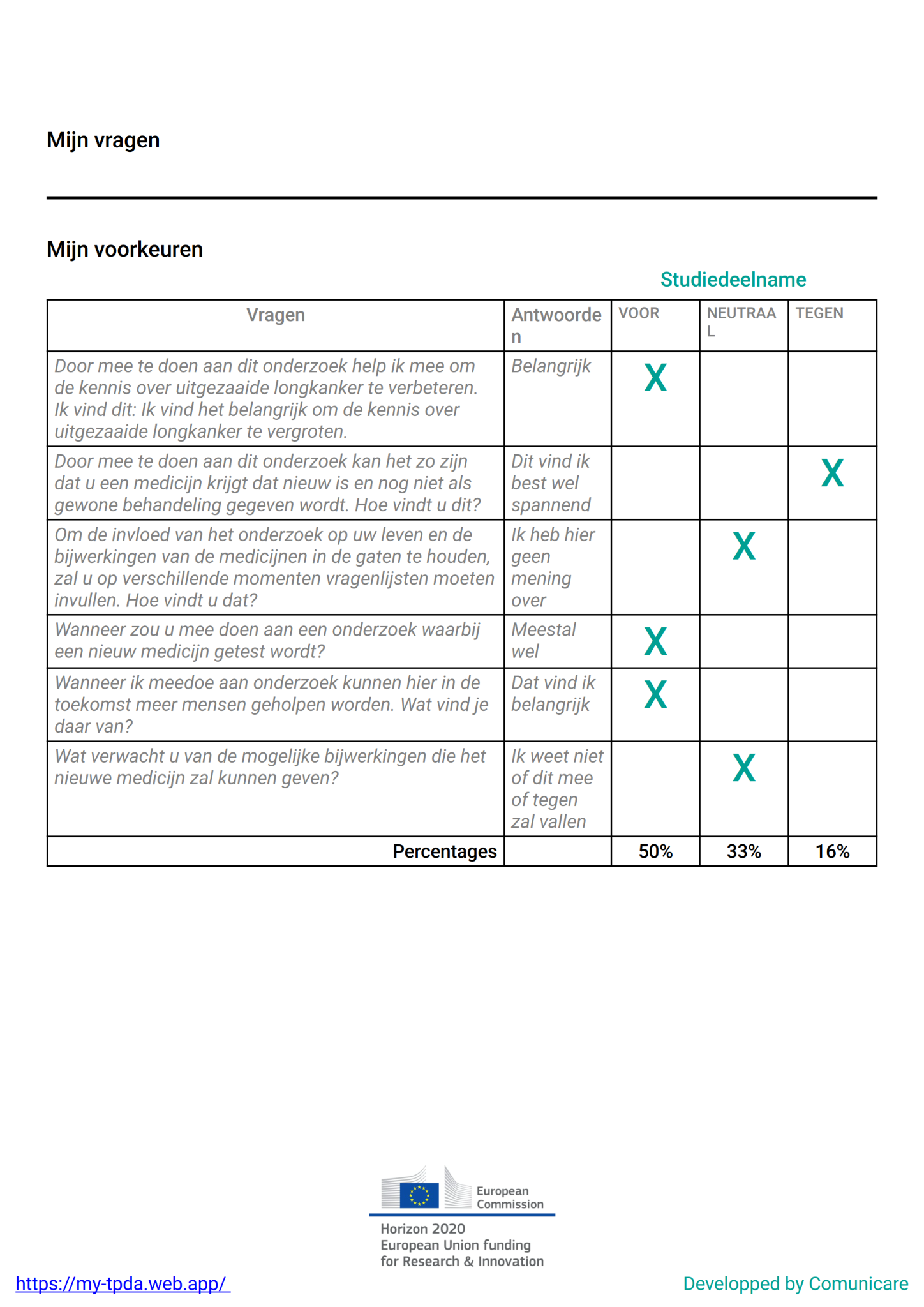 |
| --- | --- |

Figure S5: A screenshot of a PDF report that the patient can download or send by email for their meeting with the treating physician. The report contains a summary of their preferences as determined by their answers to the values clarification questions.
